# Nanoparticle-Mediated Delivery of Satraplatin to Overcome Cisplatin Drug Resistance

**DOI:** 10.3390/jfb14070387

**Published:** 2023-07-22

**Authors:** Xiaohan Jiang, Qiang Yang, Ruogu Qi, Lesan Yan

**Affiliations:** 1 State Key Laboratory of Advanced Technology for Materials Synthesis and Processing, Biomedical Materials and Engineering Research Center of Hubei Province, Wuhan University of Technology, Wuhan 430070, China; 2 Department of Obstetrics and Gynecology, Union Hospital, Tongji Medical College, Huazhong University of Science and Technology, Wuhan 430022, China; 3 School of Medicine and Holistic Integrative Medicine, Nanjing University of Chinese Medicine, Nanjing 210023, China

**Keywords:** drug resistance, drug delivery system, platinum, antitumor, satraplatin

## Abstract

Drug resistance and cancer metastasis are the major obstacles for widely used platinum-based chemotherapy. It is acknowledgement that the decreasing intracellular accumulation of anticancer drugs and increasing sulfur-binding detoxification are two major mechanisms related to drug resistance. Herein, we developed a practical and straightforward method for formulating the clinically used anticancer drug satraplatin (JM-216) with D-α-tocopheryl polyethylene glycol succinate (TPGS)-based polymers to create satraplatin-loaded nanoparticles (SatPt-NPs). The experimental results demonstrate that SatPt-NPs exhibited comparable efficacy to A2780 in treating the A2780 cisplatin-resistant ovarian cancer cell line (A2780DDP), indicating their significant potential in overcoming drug resistance. Additionally, buthionine sulfoximine (BSO) is capable of depleting intracellular glutathione (GSH), resulting in reduced detoxification. After BSO treatment, the IC_50_ value of SatPt-NPs changed from 0.178 to 0.133 μM, which remained relatively unchanged compared to cisplatin. This suggests that SatPt-NPs can overcome drug resistance by evading GSH detoxification. Therefore, SatPt-NPs have the ability to inhibit drug resistance in tumor cells and hold tremendous potential in cancer treatment.

## 1. Introduction

Ovarian cancer is one of the most common gynecologic neoplasms, accounting for a fifth of cancer mortality cases among women [1]. It has been reported to have only a 45% survival rate for patients with stage III and IV epithelial ovarian cancer (EOC), despite surgical treatment. Pertaining to chemotherapy for ovarian cancer, platinum (II)-based drugs such as cisplatin and carboplatin have been acknowledged to be the most potent therapeutic drugs [2,3,4,5,6]. Nevertheless, it has encountered severe drug resistance in clinical ovarian cancer treatment [7,8]. The underlying molecular mechanisms of platinum drug resistance have been elucidated, such as reduced drug accumulation and enhanced efflux, increasing drug detoxification by glutathione (GSH) and metallothioneins (MTs), DNA repair, and altered apoptotic signaling pathway [9,10,11].

Satraplatin (bis-(acetato)-ammine dichloro-(cyclohexylamine) platinum (IV), JM216) is a fourth-generation platinum drug, which has entered phase II and III trials and has been determined to be exceedingly potent, and is also supplemented with other chemotherapeutic drugs for the treatment of a spectrum of intractable cancers [12]. Satraplatin is known to possess two axial acetate groups that render it relatively lipophilic, which gives rise to improved permeability across cytoplasm membranes, demonstrating appreciable antitumor activity against diverse tumors, including ovarian, cervical, and lung cancers [13,14,15]. Notably, satraplatin has also shown to be highly potent toward cisplatin-resistant tumors. This may be as a result of, as opposed to, platinum (II) such as cisplatin, the oxidation state of satraplatin (platinum (IV)), imparting excellent chemical inertness and stability, which minimizes the occurrence of the GSH-mediated detoxification that is found in several Pt drugs [16,17]. Satraplatin can be facile metabolized to its platinum (II) counterpart JM-118 in cancerous cells to form Pt-DNA adducts with inter- and intra-strand crosslinks, similar to cisplatin [18]. Pt-DNA adducts induced by satraplatin can lead to inhibition of both DNA replication and transcription, as well as exert signal transduction for cell cycle arrest and apoptosis. Compared to cisplatin, the unique asymmetrical stable cyclohexane ligands of satraplatin can stabilize DNA adducts, which results in an increased inhibitory effect on translational DNA synthesis [19,20]. Meanwhile, the DNA-mismatch repairing mechanism is determined to be difficult [21,22], which consequently contributes to the overcoming of drug resistance. Although satraplatin is effective on many cisplatin-resistant cancer cells, satraplatin also displays some drawbacks such as adverse side effects, most frequently noncumulative myelosuppression (grade II–III neutropenia, thrombocytopenia, and anemia), nausea, vomiting, and diarrhea due to its immature reduction.

Currently, various approaches are being explored to overcome resistance to platinum drugs [23]. These include developing novel platinum complexes with spatial site blockers to hinder the detoxification process involving intracellular glutathione (GSH); searching for new resistance-modulating materials that can be used as adjuvants in platinum drug therapy; combining treatment with other drugs specifically targeting tumor cells; investigating the molecular-level mechanisms of platinum drugs and exploring novel proteins or signaling pathways associated with their mode of action. Among these approaches, specific nanoparticles have been chosen as nanocarriers for platinum drugs to ensure drug stability while simultaneously suppressing platinum drug resistance. Therefore, to improve the efficacy and safety of satraplatin, it is valid to develop a targeted delivery system to protect satraplatin from premature reduction and to maximize drug internalization into cancerous cells [24,25].

**Scheme 1:**
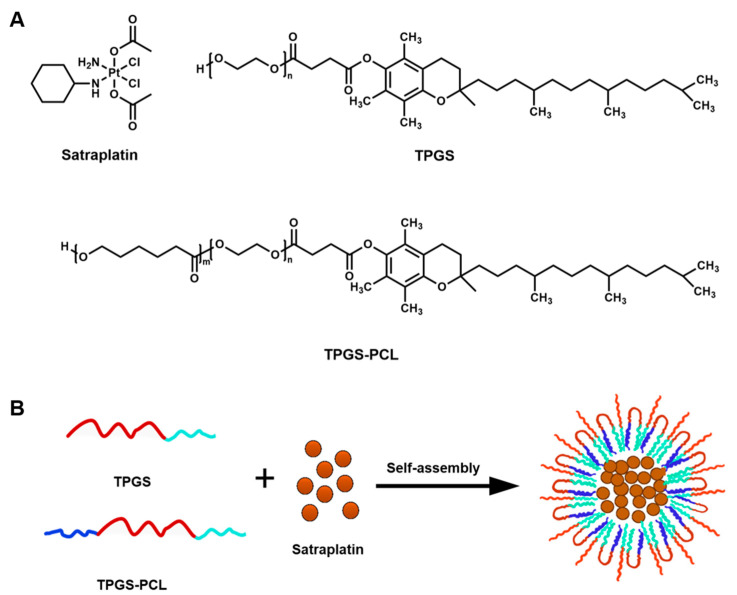
(**A**) Chemical structures of satraplatin, TPGS, and TPGS–PCL. (**B**) Schematic diagram to illustrate satraplatin-loaded nanoparticles.

It is well known that D-α-tocopheryl polyethylene glycol succinate (TPGS) is an FDA-approved surfactant that is widely utilized in the food and drug industry. TPGS is also extensively used in the construction of drug delivery carriers for hydrophobic drugs to improve their water solubility and drug delivery efficiency to tumors [26,27]. In the present study, TPGS was chosen to encapsulate satraplatin to formulate nanoparticle delivery systems (SatPt-NPs) with PEG as the hydrophilic shell and vitamin E of TPGS and satraplatin as the core (Scheme 1). To further increase the colloidal stability of the nanoparticles, TPGS–PCL was also introduced for self-assembly. We found that SatPt-NPs had a mean diameter narrowly distributed around 151 nm by DLS. *In vitro* studies have demonstrated that SatPt-NPs can overcome cisplatin resistance. In addition, a possible mechanism underpinning SatPt-NPs in overcoming cisplatin resistance is elucidated.

## 2. Materials and Methods

### 2.1. Materials

Satraplatin was purchased from Kunming Precious Metal Co., Ltd (Kunming, Yunnan, China). Vitamin E TPGS (d-alpha tocopheryl polyethylene glycol 1000 succinate) was purchased from Sigma-Aldrich (Saint Louis, MI, USA). 3-(4,5-dmethylthiazol-2-yl)-2,5-diphenyltetrazolium bromide (MTT), 4′,6-diamidino-2-phenylindole (DAPI), and rhodamine were also purchased from Sigma-Aldrich (Saint Louis, MI, USA).

### 2.2. General Methods

An inductively coupled plasma optical emission spectrometer (ICP-OES, iCAP 6300, Thermo Scientific, Waltham, MA, USA) was applied to characterize the total platinum content in the SatPt-NPs and samples in the drug-releasing experiments. An inductively coupled plasma mass spectrometer (ICP-MS, X-series II, Thermo Scientific, USA) was also used for the quantitative determination of platinum. The morphology of the NPs was observed on a JEOL JEM-1011 transmission electron microscope (Tokyo, Japan). Proton spectra of the copolymer were recorded at 400 MHz with a Bruker 400 Avance III instrument (Zurich, Switzerland). Gel permeation chromatography (GPC) measurement was performed on an Agilent 1210 LC system (Santa Clara, CA, USA) with a KD-806M GPC column in series at 25 °C with a flow rate of 1 mL/min. Tetrahydrofuran (THF) was used as the elution phase.

### 2.3. Synthesis of TPGS–PCL Copolymers

TPGS–PCL copolymers were prepared by ring-opening polymerization of ε-caprolactone monomer with vitamin E TPGS as an initiator with stannous octoate. In brief, qualitative ε-caprolactone, TPGS, and 1 wt% stannous octoate were added into a round-bottom flask. Then, the mixture was sealed and reacted in an oil bath at 120 °C for 24 h. After this, the reaction product was precipitated in excess cold methanol to remove unreacted monomer. The precipitate product was filtration-collected and dried for two days in a vacuum.

### 2.4. Preparation and Formulation of SatPt-NPs and Rho-NPs

SatPt-NPs were prepared through the nanoprecipitation method. Briefly, TPGS, TPGS–PCL, and satraplatin were mixed and dissolved in 1 mL of DMF, then 10 mL of water was added drop-wise to the flask to form a SatPt-NP solution. Then, the solution was dialyzed against water to remove excess DMF and freeze-dried to obtain lyophilized SatPt-NPs. Rhodamine-encapsulated NPs were prepared via a similar method. The diameter, PDI, and zeta potential of the SatPt-NPs were characterized with a ZS90 Nanosizer with a 633 nm He-Ne laser (Malvern, Worcestershire, UK). The platinum content of the nanoparticles was obtained by ICP-OES. The morphology of representative SatPt-NPs was observed by a JEOL JEM-1011 transmission electronic microscopy (Tokyo, Japan).

### 2.5. Drug Release from SatPt-NPs

A total of 50 mg of SatPt-NPs were suspended in 15 mL of phosphate buffer solution (pH 7.4). The solution was then placed into a dialysis bag (cutoff of 3.5 kDa) and submerged in 50 mL of PBS. The dialysis was conducted at 37 °C in an incubator. Each time, 1 mL of the sample was taken from the incubation medium at specified time intervals. After this, an equal volume of fresh PBS was added and the same procedure was performed for the releasing experiments in acetate buffer solution (pH 5.0). The release of platinum from the SatPt-NPs was measured by ICP-OES. Each of the released drugs from the SatPt-NPs was demonstrated as the percentage of the cumulative drug outside the dialysis bag to the original total drug.

### 2.6. Cell Use and Cell Culture Conditions

A2780 (human ovarian cancer, cisplatin-sensitive) and A2780DDP (human ovarian cancer, cisplatin-resistant) were purchased from the Institute of Biochemistry and Cell Biology, Chinese Academy of Sciences, Shanghai, China, and cultured in RPMI-1640 (Life Technologies, Gaithersburg, MD, USA) with 10% fetal bovine serum (Life Technologies) and 0.03% L-glutamine in 5% CO_2_ at 37 °C.

### 2.7. Evaluation of the Antitumor Efficacy of SatPt-NPs on A2780 and A2780DDP Cells

A2780 and A2780DDP cells were seeded onto a 96-well plate at 3000 cells/well density with RPMI-1640 and incubated overnight. The medium was then substituted with the medium containing various drug formulations of cisplatin, satraplatin, and SatPt-NPs. All of the drugs had platinum, and the concentrations of the drugs were modulated to a final equivalent Pt concentration from 0.0064 to 10 μM. The incubation of each drug continued for 72 h. Then, 20 μL of a 5 mg/mL PBS solution of MTT (3-(4,5-dmethylthiazol-2-yl)-2,5-diphenyltetrazolium bromide) was added onto the plates and incubated for another 4 h at 37 °C, followed by removal of the culture medium containing MTT and addition of 150 μL of DMSO to each well. Finally, the plates were shaken for 10 min, and the 570 nm UV-Vis absorbance of samples was measured by a microplate reader.

### 2.8. Intracellular Uptake of SatPt-NPs

A2780 and A2780DDP were seeded onto six-well plates at a density of 10^6^ cells per well. After this, the cells were treated with cisplatin, satraplatin, and SatPt-NPs with the final Pt concentration in the medium regulated to 10 μM and incubated at 37 °C for 1, 2, and 6 h. Next, the cells were washed five times with ice-cold PBS to remove surface-bound drugs, then incubated with 1.5 mL of 0.15 M sodium chloride (pH was adjusted to 3.0 by acetic acid) for 3 min at 4 °C, rinsed into 2 mL of cold PBS, and trypsinized. After this, the cells were counted and collected. The collected cells were treated with 1 mL of 30% H_2_O_2_/70% HNO_3_ overnight and then subjected to an ICP-MS test by diluting the desirable number of times. The uptake of drugs is described as “ng Pt/million” cells.

### 2.9. Intracellular Uptake of SatPt-NPs

A2780DDP cells were plated on coverslips in six-well plates (1 × 10^5^ cells/well) and cultured with RPMI 1640 containing 10% FBS for 24 h. The cells were then incubated with rhodamine-loaded SatPt-NPs with a fixed rhodamine concentration of 5 µM for 4 h. After removal of the media, the cells were then washed twice with cold PBS (pH 7.4) and fixed with 4% formaldehyde. Samples were later incubated with 1 mg/mL DAPI for 15 min in PBS to label the cell nucleus. The slides were mounted on coverslips and observed by using an Olympus FV1100 laser confocal scanning microscope imaging system (Tokyo, Japan).

### 2.10. Inhibition of the Cell Endocytosis of SatPt-NPs

A2780 and A2780DDP were seeded onto six-well plates with a density of 1 × 10^6^ cells per well overnight. The cells were treated with SatPt-NPs with the final platinum concentration in the culture medium regulated to 10 μM and incubated at and 37 °C for 1 h, respectively. After this, the cells were washed five times with cold PBS and incubated with 1.5 mL of 0.15 M sodium chloride (pH was adjusted to 3.0 by acetic acid) for 3 min at 4 °C, then rinsed with 2 mL of cold PBS and harvested by trypsinization. Next, the cells were counted and collected. The collected cells were treated with 1 mL of 30% H_2_O_2_/70% HNO_3_ overnight and then subjected to an ICP-MS test by diluting for desirable times. The uptake of drugs is described as “ng Pt/million” cells.

### 2.11. GSH Assay

The GSH concentration in the A2780 and A2780DDP cells was detected using a Glutathione Assay Kit (Sigma-Aldrich) following the manufacturer’s protocol. First, 10^8^ cells were transferred to a microcentrifuge tube after washing them with PBS. Second, the cells were centrifugated to obtain a cell pellet. Third, the supernatant was removed and the volume of the pellet was measured, and three times the volume of the 5% 5-sulfosalicylic acid (SSA) was added. Finally, the packed cell pellet was collected and vertexing was carried out. The solution was kept for 5 min at 2–8 °C after freezing and thawing the suspension twice, followed by centrifugation of the extract at 5000× *g* for 5 min. Lastly, the GSH concentration of the supernatant was measured on a 412 nm by Bio-raid plate reader. The GSH level in the cells is expressed as “nmol/million” cells**.**

### 2.12. MTT Assay after BSO Pretreatment

Harvested A2780 and A2780DDP cells were seeded onto 96-well plates at a density of 3000 cells/well in RPMI 1640 and incubated overnight. The 96-well plates were separated into two groups. A predetermined volume of BSO was added to the first group of plates at a final BSO concentration of 200 µM to deplete the intracellular GSH as previously reported [28]. For the second group of plates, only the same amount of PBS solution was added as controls. The two groups of plates with cells were further incubated in the incubator for another 12 h. After this, the culture media were removed, following by washing with PBS three times. Then, 100 µL of new culture media was added to the two groups of plates. The cells were then treated with various cisplatin, satraplatin, and SatPt-NP formations. All of the drugs had platinum and the drug concentrations were modulated to a final equivalent Pt concentration from 0.0064 to 10 μM. The incubation of each drug was continued for 72 h. Then, 20 μL of a 5 mg/mL PBS solution of MTT (3-(4,5-dmethylthiazol-2-yl)-2,5-diphenyltetrazolium bromide) was added to the plates and incubated for another 4 h at 37 °C, followed by removal of the culture medium containing MTT and addition of 150 μL of DMSO to each well. Finally, the plates were shaken for 10 min, and the 570 nm UV-Vis absorbance of the samples was measured by a microplate reader.

### 2.13. Cell Morphology

A2780 and A2780 DDP were seeded onto six-well plates with 1 × 10^5^ cells per well. The cells were treated with cisplatin, satraplatin, and SatPt-NPs by a fixed platinum concentration of 2.5 µM for 48 h. The morphology of the cells was observed via Nikon E100 microscopy with a digital camera (Tokyo, Japan).

### 2.14. Cell Adhesion Assay

A2780 and A2780DDP were resuspended in culture media and seeded onto 96-well tissue culture plates at a density of 2 × 10^4^ cells per well. Simultaneously, the cells were treated with cisplatin, satraplatin, and SatPt-NPs with the final platinum concentration in the culture medium regulated to 2.5 μM and incubated at 37 °C for 12 h. After this, the culture media were removed, and the cells were thoroughly washed with PBS three times. Then, fresh culture media were added and after warming up the cells in an incubator for 4 h, 20 μL of an MTT solution in PBS at a concentration of 5 mg/mL was added. The plates were incubated for another 4 h at 37 °C, followed by removal of the culture medium containing MTT and addition of 150 μL of DMSO to each well. Finally, the plates were shaken for 10 min, and the absorbance of the formazan product was measured at 570 nm by a microplate reader. The relative cell adhesion rate is expressed as the “mean absorbance values of the drug-treated cells/mean absorbance values of non-treated cells.”

### 2.15. Clone Formation

The 12-well plate-cultured A2780 and A2780 DDP cells (1 × 10^3^ cells/well) were treated with PBS, cisplatin, satraplatin, and SatPt-NPs separately at a fixed Pt dose of 2.5 μM. After seven days, formed cell clones were fixed with 4% formaldehyde and then stained with a 0.5% gentian violet PBS solution. The number of cell clones was calculated by ImageJ.

### 2.16. Statistical Analysis

The data are expressed as the mean ± standard deviation (SD). *t*-tests were applied to determine the statistical difference between the experimental and control groups. Differences were considered statistically significant when * *p* < 0.05.

## 3. Results and Discussion

### 3.1. Synthesis of a TPGS–PCL Block Copolymer

**Figure 1:**
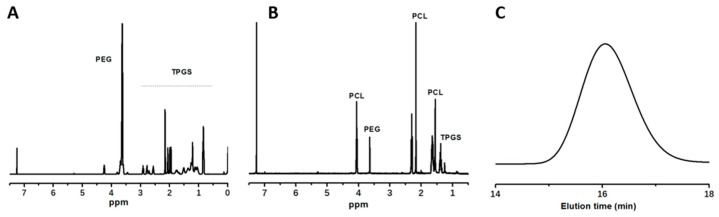
Characterizations of the synthesized polymers. ^1^H NMR spectra of TPGS (**A**) and TPGS–PCL (**B**) in CDCl_3_ and GPC of TPGS–PCL (**C**).

The TPGS–PCL copolymer was synthesized using stannous octoate as the catalyst. The synthetic schemes of vitamin E TPGS and the TPGS–PCL copolymer are shown in Scheme 1A. The chemical structure of the TPGS–PCL copolymer was characterized by ^1^H NMR in CDCl_3_. A representative ^1^H NMR spectrum of TPGS–PCL copolymer is shown in Figure 1. The peaks at 4.12, 2.33, 1.67, and 1.34 ppm can be assigned to the –CH_2_ protons of the PCL segment. Meanwhile, the peak at 3.65 ppm can be assigned to the –CH_2_ protons of the PEO components of TPGS. The multiple peaks between 1.0 and 2.0 ppm belong to the aliphatic region of the various moieties of vitamin E tails. The molecular weight of TPGS–PCL was calculated based on the ^1^H NMR measurement according to the integral area ratio between 4.12 and 3.65 ppm. As a result, the TPGS–PCL number-averaged molecular weight based on ^1^H NMR was determined to be approximately 14,500 Da.

The TPGS–PCL copolymer was further confirmed by GPC analysis. Figure 1C shows the unimodal molecular weight distribution as a narrow peak for the yielded copolymer. The polydispersity of the copolymer was determined to be approximately 1.20. The number-averaged molecular weight calculated by GPC was approximately 15,600 Da, in good accordance with the estimation based on the ^1^H NMR measurement.

### 3.2. SatPt-NP Preparation and Characterization

**Figure 2:**
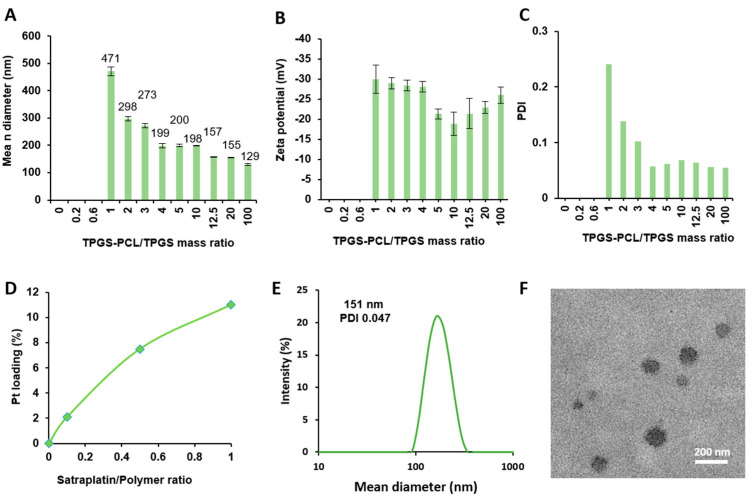
Formulation of satraplatin-loaded nanoparticles. To identify the best nanoparticle formulations for encapsulating satraplatin, varied weight ratios of TPGS–PCL/TPGS were prepared for exhaustive measurement of the diameter, zeta potential, and polydispersity index, shown as the mean diameter (**A**), zeta potential (**B**), and polydispersity index (**C**) of the nanoparticles versus the feed ratio of TPGS–PCL/TPGS. The weight ratio of TPGS–PCL/TPGS was kept at 20 by adding different ratios of satraplatin/polymer, and the Pt loading of the nanoparticles obtained was collected (**D**). Representative DLS (**E**) and TEM (**F**) results of the SatPt-NPs at a satraplatin/polymer ratio of 1.

The amphiphile of TPGS–PCL could readily self-assemble into nanoparticles with the PCL segments as the inner core and the TPGS segments as the hydrophilic shell. Moreover, TPGS was added to stabilize the micellar formation. Micelles of varied ratios of TPGS–PCL/TPGS were prepared and the diameter, PDI, and zeta potential are characterized in Figure 2A–C, respectively. The diameter decreased from 470 to 155 nm in the range of a TPGS–PCL/TPGS ratio from 1 to 20 (Figure 2A). Nanoparticles with a narrow PDI (below 0.1) were achieved when the TPGS–PCL/TPGS ratios were above 4 (Figure 2B). All micelles at varied TPGS–PCL/TPGS ratios exhibited similar zeta potential (nearly −25 mV) (Figure 2C). The hydrophobic nature of the PCL core is capable of encapsulating hydrophobic drugs. Hydrophobic satraplatin was entrapped at the optimized TPGS–PCL/TPGS ratio of 20 to formulate SatPt-NPs. The drug-loading percentage of satraplatin in nanoparticles increased by up to 11% with rising ratios of satraplatin/NPs (Figure 2D). The diameter and PDI of SatPt-NPs at 11% drug loading were 151 nm and 0.047, respectively (Figure 2E). Representative TEM morphologies of the SatPt-NPs are shown in Figure 2F. The SatPt-NPs exhibited spherical structures through TEM with an average diameter of approximately 130–140 nm, in agreement with DLS results.

### 3.3. SatPt-NPs Showed a pH-Responsive Release Profile

It is important that SatPt-NPs can release encapsulated drugs for anticancer efficacy. Drug-releasing experiments were performed via dialysis against a buffered solution at both physiological pH 7.4 and subcellular endosomal pH 5.0. ICP-OES was applied for quantification of the released platinum. The relative released drug relative to the total drug payloads was measured as a function of release time.

**Figure 3:**
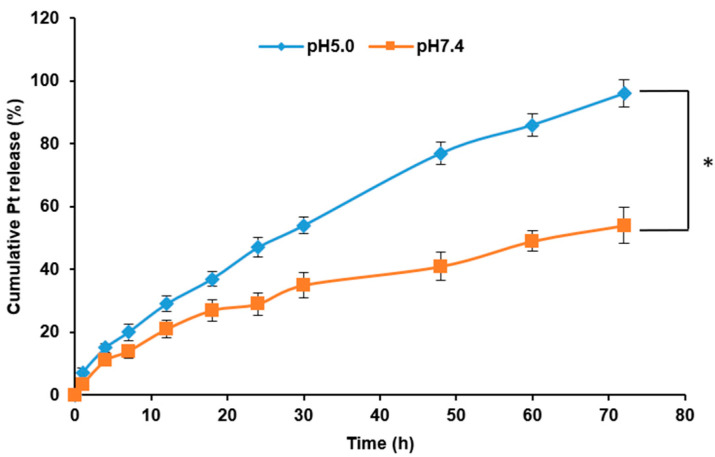
Drug-release profiles of the SatPt-NPs under incubation at pH 5.0 and pH 7.4. * *p* < 0.05.

On the release profiles (Figure 3), the following features were found: Pt was released by following a pH-dependent manner and promoted drug release was observed at pH 5.0 than pH 7.4. At 72 h, the cumulative release percentages of Pt at pH 5.0 and pH 7.4 were determined to be approximately 96% and 46%, respectively. A plausible reason for this acid-stimulated drug release is the faster hydrolysis of the ester satraplatin linkage in an acidic milieu, thus leading to breakage of the ester bond in PCL segments, leading to structural dissociation.

### 3.4. Cisplatin Resistance Overcome by SatPt-NPs

**Figure 4:**
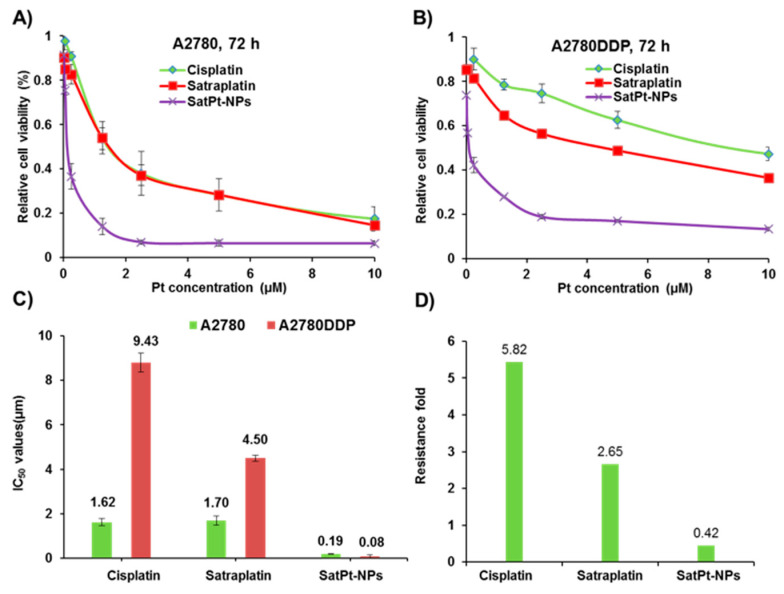
Cisplatin resistance overcome by the SatPt-NPs. *In vitro* cytotoxic evaluation of cisplatin, satraplatin, and the SatPt-NPs on A2780 (**A**) and A2780DDP cells (**B**) for 72 h. IC_50_ values (**C**) and drug resistance fold (**D**) of cisplatin, satraplatin, and SatPt-NPs on A2780 and A2780DDP cells at 72 h were calculated.

The *in vitro* antitumor efficacy of the SatPt-NPs was determined using normal A2780 and cisplatin-resistant A2780DDP ovarian cancer cells. Cell viabilities as a function of the drug concentrations of cisplatin, satraplatin, and SatPt-NPs were evaluated and summarized in Figure 4A,B. As shown in Figure 4A, cisplatin and satraplatin showed comparable potency in inhibiting cell growth up to 10 µM. Notably, the SatPt-NPs appeared to be more efficacious than both cisplatin and satraplatin. Moreover, even at the highest dosage, the cell viability of the A2780 cells treated with cisplatin and satraplatin still exceeded 20%, as opposed to merely 6.5% for the SatPt-NPs. In clinical treatment, small populations of cancer cells that survive chemotherapy contributed to the cancer recurrence. The above results indicate the overwhelming potency of SatPt-NPs. Furthermore, we tested the SatPt-NPs on cisplatin-resistant A2780DDP cells. Cell viabilities as a function of drug concentrations are shown in Figure 4B (cisplatin and satraplatin as controls). Cisplatin was determined to be the least potent on A2780DDP cells. On the contrary, the SatPt-NPs proved to be the most potent as the cell viabilities appeared to be subjected to a sharp drop with a rising dosage. At the highest dosage of 10 µM, the cell viability of cisplatin, satraplatin, and the SatPt-NPs was determined to be approximately 47%, 36%, and 13%, respectively.

To obtain a quantitative insight, the IC_50_ values of cisplatin, satraplatin, and the SatPt-NPs on A2780 and A2780DDP cells are summarized in Figure 4C. As previously described, satraplatin, and SatPt-NPs are more potent than cisplatin. The results in Figure 4C highlight that cisplatin had an IC_50_ index of 1.62 and 8.80 µM on A2780 and A2780DDP cells, respectively, while satraplatin had IC_50_ index of 1.70 and 4.50 µM and the SatPt-NPs had IC_50_ values of 0.19 and 0.08 µM, respectively. Therefore, the SatPt-NPs were validated to exert a significantly higher antitumor efficacy on both A2780 and A2780DDP cells, indicating the appreciable translation of SatPt-NPs into clinical applications. Moreover, a drug-resistant fold for each drug was defined: IC_50_ on A2780DDP/IC_50_ on A2780 of a specific drug. The results are summarized in Figure 4D. Previous results showed that cisplatin and satraplatin exhibit similar efficiency in the A2780 cell line (Figure 4A). Yet, cisplatin and satraplatin had 5.43- and 2.65-fold lower efficiency on A2780 DDP than A2780, respectively. Notably, the SatPt-NPs exhibited comparable efficacy on A2780DDP to A2780, with a 0.44-fold IC_50_ value, indicating its appreciable properties in overcoming drug resistance.

### 3.5. SatPt-NPs Overcome Resistance by Maximizing Drug Internalization

**Figure 5:**
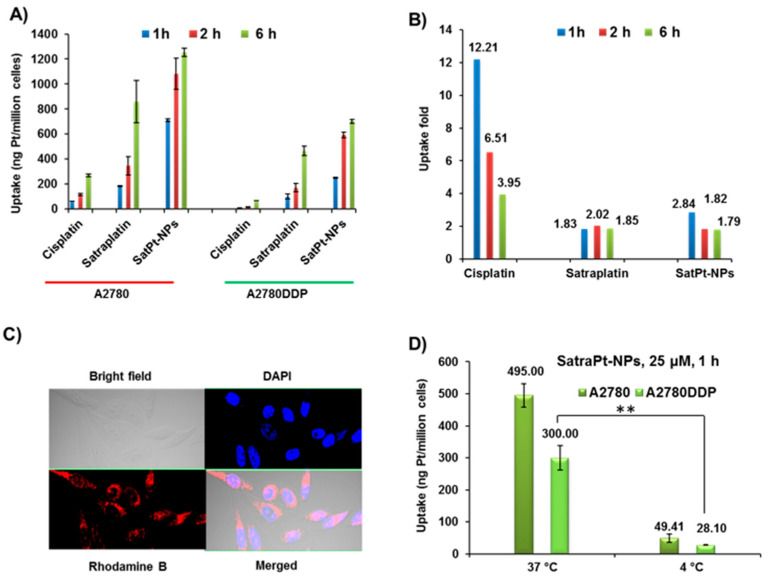
SatPt-NPs overcome drug resistance by maximizing drug internalization. The platinum uptake (**A**) and relative drug uptake fold (**B**) of A2780DDP cells compared to the drug uptake of A2780 after 1, 2, and 6 h of drug treatment. Data were normalized by the uptake of cisplatin at 1 h for A2780 cells which was set as 1; typical cellular uptake by Rh B-loaded NPs (**C**). Uptake of SatPt-NPs at 4 °C was substantially inhibited, indicating an energy-dependent internalization pathway (**D**). ** *p* < 0.01.

In general, the efficacy of cisplatin is majorly determined by its intracellular drug amount [29]. Pt drug internalization is considered an important factor for determining drug efficacy. The intracellular level of cisplatin, satraplatin, and SatPt-NPs at the same dosage of Pt (10 µM) by A2780 and A2780DDP cells at 1, 2, and 6 h was measured by ICP-MS. This provides important insight into how SatPt-NPs can overcome cisplatin resistance. As shown in Figure 5A, some important features exist for the uptake assay. For both A2780 and A2780DDP cells, the uptake of all drugs, including cisplatin, satraplatin, and SatPt-NPs, was increased from 1 to 6 h. Specifically, the progressive uptake of SatPt-NPs at 1, 2, and 6 h by A2780 cells was observed to reach from 711 and 1081 ng Pt/million cells to 1253 ng Pt/million cells, while this value changed to 250, 593, and 702 ng Pt/million cells for A2780DDP cells in the same period of time. Cisplatin and satraplatin also displayed similar changes. For the same drug at the same time point, less drug was determined to internalized into A2780DDP cells compared to A2780 cells. To provide better insight into this, the “reduced uptake fold” was determined for each drug by calculating the drug uptake by A2780 and A2780DDP cells. The results are shown in Figure 5B. From 1 to 6 h, the uptake fold of cisplatin was highest, though it decreased from 12.2 to 3.95. Unlike cisplatin, satraplatin showed an uptake fold of around 2.0 over this time and no obvious change was observed. However, the uptake fold for the SatPt-NPs was the lowest and decreased from 2.84 to 1.79 from 1 to 6 h. The limited difference in uptake of SatPt-NPs on A2780 and A2780DDP cells suggests that SatPt-NPs may be internalized to the cancer cells via a differing pathway from cisplatin and satraplatin. Taken together, cells treated with the SatPt-NPs maximized the amount of drug internalized, while the reduced uptake fold at different time points was minimized. Here, the unique uptake properties of SatPt-NPs may explain how they overcome cisplatin drug resistance.

Nanoparticles are believed to be endocytosed by cancerous cells. We prepared rhodamine-encapsulated Sat-NPs to track the endocytosis of the nanoparticles by A2780 DDP. As shown in Figure 5C, the red dots expanded in the cancer cells, which represents effective endocytosis of the Sat-NPs in drug-resistant cancer cells. To further investigate SatPt-NP endocytosis by A2780 and A2780DDP cells, we compared the intracellular uptake of the SatPt-NPs at 37 and 4 °C. Note that incubation of cells at low temperatures reduces the metabolism of cells, thus inhibiting energy-dependent endocytosis. As expected, the uptake of SatPt-NPs at 4 °C was reduced, as shown in Figure 5D. At 37 °C, the uptake of SatPt-NPs was 495 and 300 ng Pt/million cells for A2780 and A2780DDP, respectively, while at 4 °C, the values were 49.41 and 28.10 ng Pt/million cells for A2780 and A2780DDP, respectively. This is an almost 10-fold reduction in uptake on both cell lines from 37 to 4 °C, which demonstrates that the uptake of SatPt-NPs was based on the energy-dependent endocytic pathway.

Taken together, we can clearly show here that SatPt-NPs can adopt endocytosis to internalize drugs, which is important in circumventing the cisplatin cellular uptake pathway and overcoming drug resistance.

### 3.6. SatPt-NPs Overcome Resistance by Minimizing GSH Detoxification

**Figure 6:**
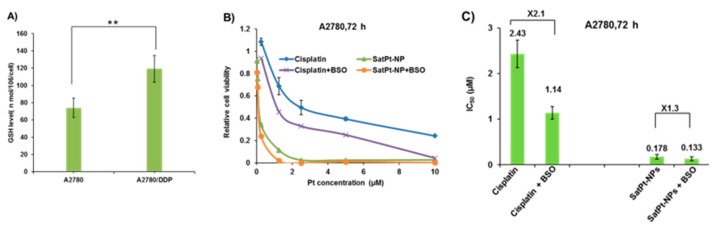
SatPt-NPs overcome cisplatin resistance by improved inertness to GSH-mediated detoxification. (**A**) GSH levels in A2780 and A2780DDP cells; (**B**) dose-dependent cell viability of cisplatin and SatPt-NPs with and without GSH depletion by BSO (200 µM) for 12 h and an additional 72 h of incubation; (**C**) IC_50_ values of cisplatin and SatPt-NPs with and without GSH depletion by BSO (200 µM). ** *p* < 0.01.

It is extensively reported that cisplatin-resistant cell lines have a higher expression of GSH, which is responsible for detoxification of Pt drugs in cells [30,31]. For the case of ovarian cancer, we tested the expression of GSH levels in A2780 and A2780DDP cells via a GSH assay. The results in Figure 6A show that A2780DDP cells have 1.5-fold more GSH than A2780 cells, in accordance with previously reported results [32]. This high expression of GSH contributes to cisplatin resistance in A2780DDP cells. The chemical inertness of satraplatin and further encapsulation within nanoparticles is speculated to eventually contribute to more inert responsiveness to GSH fluctuations in cancer cells and thus decreased resistance. To test this speculation, cells were pretreated with 200 µM of BSO for 12 h, which can deplete the GSH levels in cancer cells by >90% [33]. The cells were treated with cisplatin and SatPt-NPs. Cells pre-treated with PBS were used as controls. Representative cell viability with or without pretreated of BSO for each drug on A2780 was evaluated. As shown in Figure 6B, the cell viabilities of both cisplatin and the SatPt-NPs shifted down if BSO pretreatment was performed, indicating enhancement of the drug efficacy. IC_50_ values were collected in Figure 6C (A2780). After treatment with BSO, the IC_50_ values of cisplatin changed from 2.43 to 1.14 µM, while the IC_50_ values of the SatPt-NPs changed from 0.178 to 0.133 µM. Most likely, BSO was able to deplete the intracellular GSH, leading to reduced detoxification. However, it is obvious that the enhancement in efficacy was greater for cisplatin than for the SatPt-NPs (2.1- to 1.3-fold with or without treated with BSO). In other words, the SatPt-NPs were much more inert to GSH reduction, which could further explain how they overcome drug resistance by escaping GSH detoxification.

### 3.7. SatPt-NPs Inhibit Cell Adhesion and Colony Formation

**Figure 7:**
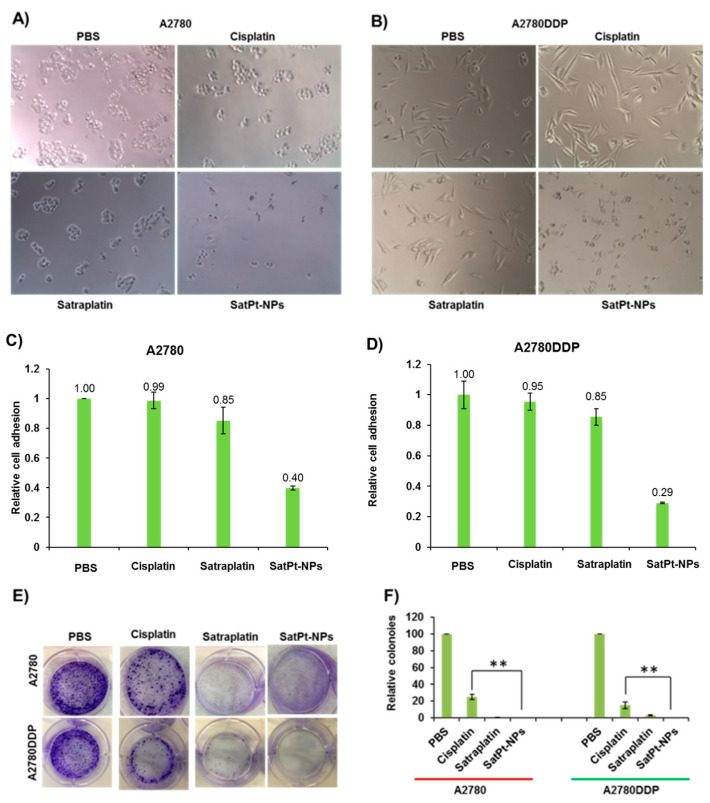
SatPt-NPs induce cell morphology changes and inhibit cell adhesion and colony formation. Cell morphology changes after treatment with cisplatin, satraplatin, and SatPt-NPs at an equivalent drug concentration of 2.5 µM based on Pt for 4 h on A2780 (**A**) and A2780DDP cells (**B**). Treatment of the cells with SatPt-NPs can reduce cancer cell adhesion (**C**,**D**) and colony formation (**E**,**F**) on both A2780 and A2780DDP cells. ** *p* < 0.01.

Under low-dose cisplatin treatment, significant morphological changes were not evident in A2780DDP cells, whereas a substantial number of contracted cells were observed in A2780 cells. However, noticeable morphological changes were observed when both A2780 and A2780DDP cells were treated with SatPt-NPs at the same dosage as cisplatin. Following SatPt-NP treatment, A2780 and A2780DDP cells exhibited deformation (Figure 7A,B), with a significant increase in the proportion of round cells. During the experimental process, cells were observed to eventually detach from the culture dish, exhibiting cytoplasmic vacuolization and cellular fragmentation. Therefore, treatment with SatPt-NPs induces morphological changes in cells, which may further lead to cell apoptosis.

It is reported that cell–matrix interactions result in cytoskeletal reorganization and the activation of multiple signal transduction pathways that directly influence cell survival, growth, and differentiation. Moreover, cell adhesion to a single matrix is sufficient to inhibit the apoptosis induced by mechanistically distinct cytotoxicity, which leads to drug resistance [34,35]. Therefore, cell adhesion inhibition is a key factor in overcoming drug resistance chemotherapy. We tested the ability of SatPt-NPs to inhibit the cell adhesion of A2780 and A2780DDP cells. Cells were seeded onto 96-well tissue culture plates and cisplatin, satraplatin, and SatPt-NPs at a Pt concentration of 2.5 µM were added simultaneously. At 12 h post-incubation, the cells were thoroughly washed with cold PBS and the relative cell adhesion to the plates as compared to PBS-treated cells was measured by MTT assay after another 4 h of incubation. The results are shown in Figure 7C (A2780) and Figure 7D (A2780DDP). Cisplatin showed almost no cell adhesion inhibition (<5%). Notably, the SatPt-NPs showed the greatest inhibition on both cell lines (60% on A2780 and 71% on A2780DDP) compared to only 15% cell adhesion inhibition for satraplatin on both A2780 and A2780DDP. The results here demonstrate that SatPt-NPs have high potency in overcoming drug resistance through inhibiting cell adhesive ability. Moreover, we performed a colony formation assay on the A2780 and A2780 DDP cancer cell lines, which can present the effect of drugs on proliferating tumor cells. As shown in Figure 7E,F, cells treated with cisplatin demonstrated plenty of colonies. However, cells treated with satraplatin showed both a lower colony number and a smaller size owing to enhanced growth inhibition. Remarkably, almost no colony formation was achieved for both A2780 and A2780 DDP cells upon treatment with SatPt-NPs, again demonstrating the intriguing potency of SatPt-NPs.

## 4. Conclusions

In summary, the present study provided the first example of using nanoparticles to deliver satraplatin, which is an important chemotherapeutic Pt(IV) prodrug that has entered clinical trials. Importantly, manufactured SatPt-NPs can overcome cisplatin resistance in contrast to satraplatin itself. We found that SatPt-NPs can overcome drug resistance by means of maximizing drug uptake into both A2780 and A2780DDP cell lines and minimizing GSH-mediated detoxification. Furthermore, SatPt-NPs can internalize drugs through endocytosis, thereby achieving efficient drug delivery. A preliminary *in vitro* cell adhesion assay revealed that SatPt-NPs have remarkable inhibitory potency in cell adhesion, which is also a key process in drug resistance that results in therapy failure in the clinic. Altogether, SatPt-NPs possess the capability to suppress drug resistance in tumor cells and exhibit significant potential in cancer treatment. Additionally, through the modification and functionalization of the nanocarrier, a novel drug delivery system can be developed, providing advantages in targeted delivery and overcoming tumor resistance.

## Data Availability

Data are available upon request from the corresponding author.

## References

[1] Stewart C., Ralyea C., Lockwood S. (2019). Ovarian Cancer: An Integrated Review. Semin. Oncol. Nurs..

[2] Xiao H., Yan L., Dempsey E.M., Song W., Qi R., Li W., Huang Y., Jing X., Zhou D., Ding J. (2018). Recent progress in polymer-based platinum drug delivery systems. Prog. Polym. Sci..

[3] Li W., Jiang M., Cao Y., Yan L., Qi R., Li Y., Jing X. (2016). Turning Ineffective Transplatin into a Highly Potent Anticancer Drug via a Prodrug Strategy for Drug Delivery and Inhibiting Cisplatin Drug Resistance. Bioconjug. Chem..

[4] Song H., Kang X., Sun J., Jing X., Wang Z., Yan L., Qi R., Zheng M. (2016). Nanoparticle delivery of sterically hindered platinum(IV) prodrugs shows 100 times higher potency than that of cisplatin upon light activation. Chem. Commun. (Camb.).

[5] Song H., Li W., Qi R., Yan L., Jing X., Zheng M., Xiao H. (2015). Delivering a photosensitive transplatin prodrug to overcome cisplatin drug resistance. Chem. Commun. (Camb.).

[6] Zhang X., Feng C., Jing X., Yan L. (2023). A versatile method to deliver platinum (II) drugs via thiol-ene/yne click reaction of polypeptide. Eur. Polym. J..

[7] Rottenberg S., Disler C., Perego P. (2021). The rediscovery of platinum-based cancer therapy. Nat. Rev. Cancer.

[8] Tsvetkova D., Ivanova S. (2022). Application of Approved Cisplatin Derivatives in Combination Therapy against Different Cancer Diseases. Molecules.

[9] Holford J., Sharp S.Y., Murrer B.A., Abrams M., Kelland L.R. (1998). *In vitro* circumvention of cisplatin resistance by the novel sterically hindered platinum complex AMD473. Br. J. Cancer.

[10] Goddard P., Valenti M., Kelland L.R. (1994). The role of glutathione (GSH) in determining sensitivity to platinum drugs *in vivo* in platinum-sensitive and -resistant murine leukaemia and plasmacytoma and human ovarian carcinoma xenografts. Anticancer Res..

[11] Wu F., Du Y., Yang J., Shao B., Mi Z., Yao Y., Cui Y., He F., Zhang Y., Yang P. (2022). Peroxidase-like Active Nanomedicine with Dual Glutathione Depletion Property to Restore Oxaliplatin Chemosensitivity and Promote Programmed Cell Death. ACS Nano.

[12] Doshi G., Sonpavde G., Sternberg C.N. (2012). Clinical and pharmacokinetic evaluation of satraplatin. Expert Opin. Drug Metab. Toxicol..

[13] Choy H. (2006). Satraplatin: An orally available platinum analog for the treatment of cancer. Expert Rev. Anticancer Ther..

[14] van Dorp E.L.A., Yassen A., Dahan A. (2007). Naloxone treatment in opioid addiction: The risks and benefits. Expert Opin. Drug Saf..

[15] Kelland L. (2007). Broadening the clinical use of platinum drug-based chemotherapy with new analogues: Satraplatin and picoplatin. Expert Opin. Investig. Drugs.

[16] Zheng Y.-R., Suntharalingam K., Johnstone T.C., Yoo H., Lin W., Brooks J.G., Lippard S.J. (2014). Pt(IV) Prodrugs Designed to Bind Non-Covalently to Human Serum Albumin for Drug Delivery. J. Am. Chem. Soc..

[17] Frezza M., Hindo S., Chen D., Davenport A., Schmitt S., Tomco D., Dou Q.P. (2010). Novel Metals and Metal Complexes as Platforms for Cancer Therapy. Curr. Pharm. Des..

[18] Yuan X., Zhang W., He Y., Yuan J., Song D., Chen H., Qin W., Qian X., Yu H., Guo Z. (2020). Proteomic analysis of cisplatin- and oxaliplatin-induced phosphorylation in proteins bound to Pt-DNA adducts. Metallomics.

[19] McKeage M.J. (2005). New-generation platinum drugs in the treatment of cisplatin-resistant cancers. Expert Opin. Investig. Drugs.

[20] Sarkisyan Z.M., Shkutina I.V., Srago I.A., Kabanov A.V. (2022). Relevance of Using Platinum-Containing Antitumor Compounds (A Review). Pharm. Chem. J..

[21] Choy H., Park C., Yao M. (2008). Current status and future prospects for satraplatin, an oral platinum analogue. Clin. Cancer Res..

[22] Todd R.C., Lippard S.J. (2009). Inhibition of transcription by platinum antitumor compounds. Metallomics.

[23] Xie P., Wang Y., Wei D., Zhang L., Zhang B., Xiao H., Song H., Mao X. (2021). Nanoparticle-based drug delivery systems with platinum drugs for overcoming cancer drug resistance. J. Mater. Chem. B.

[24] Kang X., Xiao H.H., Song H.Q., Jing X.B., Yan L.S., Qi R.G. (2015). Advances in drug delivery system for platinum agents based combination therapy. Cancer Biol. Med..

[25] Johnstone T.C., Suntharalingam K., Lippard S.J. (2016). The Next Generation of Platinum Drugs: Targeted Pt(II) Agents, Nanoparticle Delivery, and Pt(IV) Prodrugs. Chem. Rev..

[26] Mi Y., Zhao J., Feng S.-S. (2012). Vitamin E TPGS prodrug micelles for hydrophilic drug delivery with neuroprotective effects. Int. J. Pharm..

[27] Yang C., Wu T., Qi Y., Zhang Z. (2018). Recent Advances in the Application of Vitamin E TPGS for Drug Delivery. Theranostics.

[28] Kurdi M., Sivakumaran V., Duhe R.J., Aon M.A., Paolocci N., Booz G.W. (2012). Depletion of cellular glutathione modulates LIF-induced JAK1-STAT3 signaling in cardiac myocytes. Int. J. Biochem. Cell Biol..

[29] Qi R., Xiao H., Wu S., Li Y., Zhang Y., Jing X. (2015). Design and delivery of camplatin to overcome cisplatin drug resistance. J. Mater. Chem. B.

[30] Chen H.H.W., Kuo M.T. (2010). Role of glutathione in the regulation of Cisplatin resistance in cancer chemotherapy. Met. Based Drugs.

[31] Peklak-Scott C., Smitherman P.K., Townsend A.J., Morrow C.S. (2008). Role of glutathione S-transferase P1-1 in the cellular detoxification of cisplatin. Mol. Cancer Ther..

[32] Okuno S., Sato H., Kuriyama-Matsumura K., Tamba M., Wang H., Sohda S., Hamada H., Yoshikawa H., Kondo T., Bannai S. (2003). Role of cystine transport in intracellular glutathione level and cisplatin resistance in human ovarian cancer cell lines. Br. J. Cancer.

[33] Lee M., Cho T., Jantaratnotai N., Wang Y.T., McGeer E., McGeer P.L. (2010). Depletion of GSH in glial cells induces neurotoxicity: Relevance to aging and degenerative neurological diseases. FASEB J..

[34] Damiano J.S., Hazlehurst L.A., Dalton W.S. (2001). Cell adhesion-mediated drug resistance (CAM-DR) protects the K562 chronic myelogenous leukemia cell line from apoptosis induced by BCR/ABL inhibition, cytotoxic drugs, and gamma-irradiation. Leukemia.

[35] Hazlehurst L.A., Dalton W.S. (2001). Mechanisms associated with cell adhesion mediated drug resistance (CAM-DR) in hematopoietic malignancies. Cancer Metastasis Rev..

